# Pharmacokinetics and safety/tolerability of isoniazid, rifampicin and pyrazinamide in children and adolescents treated for tuberculous meningitis

**DOI:** 10.1136/archdischild-2020-321426

**Published:** 2021-06-28

**Authors:** Rovina Ruslami, Fajri Gafar, Vycke Yunivita, Ida Parwati, Ahmad R Ganiem, Rob E Aarnoutse, Bob Wilffert, Jan-Willem C Alffenaar, Heda M Nataprawira

**Affiliations:** 1 Division of Pharmacology and Therapy, Department of Biomedical Sciences, Faculty of Medicine, Universitas Padjadjaran, Bandung, Indonesia; 2 Unit of PharmacoTherapy, -Epidemiology and -Economics, Groningen Research Institute of Pharmacy, University of Groningen, Groningen, The Netherlands; 3 Department of Clinical Pathology, Faculty of Medicine, Universitas Padjadjaran, Hasan Sadikin Hospital, Bandung, Indonesia; 4 Department of Neurology, Faculty of Medicine, Universitas Padjadjaran, Hasan Sadikin Hospital, Bandung, Indonesia; 5 Department of Pharmacy, Radboud Institute for Health Sciences, Radboud University Medical Center, Nijmegen, The Netherlands; 6 Department of Clinical Pharmacy and Pharmacology, University Medical Center Groningen, University of Groningen, Groningen, The Netherlands; 7 School of Pharmacy, Faculty of Medicine and Health, University of Sydney, Sydney, New South Wales, Australia; 8 Westmead Hospital, Sydney, New South Wales, Australia; 9 Division of Pediatric Respirology, Department of Child Health, Faculty of Medicine, Universitas Padjadjaran, Hasan Sadikin Hospital, Bandung, Indonesia

**Keywords:** pharmacology, therapeutics, microbiology

## Abstract

**Objective:**

To assess the pharmacokinetics and safety/tolerability of isoniazid, rifampicin and pyrazinamide in children and adolescents with tuberculous meningitis (TBM).

**Design:**

Prospective observational pharmacokinetic study with an exploratory pharmacokinetic/pharmacodynamic analysis.

**Setting:**

Hasan Sadikin Hospital, Bandung, Indonesia.

**Patients:**

Individuals aged 0–18 years clinically diagnosed with TBM and receiving first-line anti-tuberculosis drug dosages according to revised WHO-recommended treatment guidelines.

**Interventions:**

Plasma and cerebrospinal fluid (CSF) concentrations of isoniazid, rifampicin and pyrazinamide were assessed on days 2 and 10 of treatment.

**Main outcome measures:**

Plasma exposures during the daily dosing interval (AUC_0–24_), peak plasma concentrations (*C*_max_) and CSF concentrations.

**Results:**

Among 20 eligible patients, geometric mean AUC_0–24_ of isoniazid, rifampicin and pyrazinamide was 18.5, 66.9 and 315.5 hour∙mg/L on day 2; and 14.5, 71.8 and 328.4 hour∙mg/L on day 10, respectively. Large interindividual variabilities were observed in AUC_0–24_ and *C*_max_ of all drugs. All patients had suboptimal rifampicin AUC_0–24_ for TBM treatment indication and very low rifampicin CSF concentrations. Four patients developed grade 2–3 drug-induced liver injury (DILI) within the first 4 weeks of treatment, in whom anti-tuberculosis drugs were temporarily stopped, and no DILI recurred after reintroduction of rifampicin and isoniazid. AUC_0–24_ of isoniazid, rifampicin and pyrazinamide along with *C*_max_ of isoniazid and pyrazinamide on day 10 were higher in patients who developed DILI than those without DILI (p<0.05).

**Conclusion:**

Higher rifampicin doses are strongly warranted in treatment of children and adolescents with TBM. The association between higher plasma concentrations of isoniazid, rifampicin and pyrazinamide and the development of DILI needs confirmatory studies.

## Abstract (precis)

Children with TB meningitis do poorly, and this paper examines the pharmacokinetics of a commonly used drug combination to understand if drug factors contribute.

**What is already known on this topic?**

- Pharmacokinetic data of anti-tuberculosis (TB) drugs in children with tuberculous meningitis (TBM), particularly among the Indonesian paediatric population are lacking.
- Suboptimal or toxic concentrations of anti-TB drugs contribute to unfavourable treatment outcomes.

**What this study adds?**

- Suboptimal plasma exposures and very low cerebrospinal fluid concentrations of rifampicin were observed in all patients; higher doses for this pivotal drug are strongly warranted.
- The association between higher plasma concentrations of isoniazid, rifampicin and pyrazinamide and the development of drug-induced liver injury during TBM treatment needs confirmatory studies.

## Introduction

Tuberculosis (TB) remains a major global health challenge with 1.2 million new paediatric cases and >220 000 deaths in children aged <15 years.^1^ Tuberculous meningitis (TBM), as the most devastating manifestation of TB, accounts for approximately 20% of childhood TB mortality and results in neurological sequelae in more than 50% of survivors.^2 3^ Management of TBM poses continuing challenges, mainly due to the lack of understanding of the pathogenesis, a lengthy process in obtaining a definite diagnosis and suboptimal antimicrobial drug therapy.^3^ Delayed or late presentation of TBM is a major problem associated with worse outcomes.^2^

First-line anti-TB drug doses for treatment of children with TBM were revised by the WHO in 2010^4^ and are similar to those described for children with pulmonary TB (PTB).^4 5^ Following this revised dosing, sufficient plasma concentrations of isoniazid, rifampicin and pyrazinamide in children aged <2 years were reported.^6^ However, subtherapeutic concentrations are still shown in high proportions of patients, particularly among young children for rifampicin and pyrazinamide^7–9^ and among fast acetylators for isoniazid.^8^ Furthermore, rifampicin and ethambutol have poor cerebrospinal fluid (CSF) penetration,^10 11^ which in case of TBM might lead to subtherapeutic concentrations at the site of infection. As an alternative TBM treatment option in children, the WHO suggests high-dose short-course therapy using isoniazid, rifampicin and pyrazinamide, with addition of ethionamide instead of ethambutol.^5 10 12^

Pharmacokinetic (PK) data of anti-TB drugs in children with TBM are lacking. PK evaluation of first-line anti-TB drugs is important because suboptimal concentrations might lead to unfavourable outcomes such as treatment failure and death.^13^ On the contrary, exposure to supratherapeutic concentrations might play a role in increasing the risk of adverse effects, like anti-TB drug-induced liver injury (DILI).^14^ We aimed to describe PK and safety/tolerability of isoniazid, rifampicin and pyrazinamide in Indonesian children and adolescents treated for TBM.

## Methods

### Study design and population

We performed a prospective observational PK and safety/tolerability study with an exploratory pharmacokinetic/pharmacodynamic (PK/PD) analysis in children and adolescents aged ≤18 years at Hasan Sadikin Hospital, Bandung, Indonesia, from March 2018 to January 2020. Written informed consent for study participation was obtained from parents/legal guardians, with additional verbal consent/assent from competent children aged >12 years.

Initial screening among those with suspected meningitis included physical and clinical examinations, blood chemistry and haematology measurements, chest radiography and CSF analysis. Neuroradiology and microbiological examination from CSF/extraneural samples including smear microscopy for acid-fast bacilli, culture for *Mycobacterium tuberculosis* and GeneXpert MTB/RIF assay were performed, if applicable. In our setting, neuroradiology and GeneXpert testing during screening are not covered by government health insurance. Patients with definite TBM (microbiologically proven from CSF examination), and those clinically diagnosed with probable/possible TBM as determined by case definition criteria,^15^ were eligible for inclusion in this study. Our exclusion criteria were mixed bacterial meningitis, taking anti-TB drugs ≥3 days, HIV co-infection, baseline alanine/aspartate aminotransferases (ALT/AST) >3× the ULN (reference ranges for ALT: 16–63 U/L and AST: 15–37 U/L) and medical conditions not allowing for inclusion according to the attending physician (eg, ventriculoperitoneal shunt, rapid clinical deterioration, kidney disease or autoimmune disorders).

### Treatment

Treatment regimens were based on the current WHO guidelines in accordance with the Indonesian Paediatric Society guidelines for TBM treatment in children, consisting of daily isoniazid (7–15 mg/kg), rifampicin (10–20 mg/kg), pyrazinamide (30–40 mg/kg) and ethambutol (15–25 mg/kg) for a 2-month intensive phase, followed by a 10-month continuation phase of isoniazid and rifampicin at the same doses.^5 16^ All patients (including those weighing >25 kg) received dispersible fixed-dose combinations of isoniazid/rifampicin/pyrazinamide at 50/75/150 mg, with addition of ethambutol in a separate tablet. All anti-TB drugs (Kimia Farma, Indonesia) were taken orally on an empty stomach under directly observed treatment. For unconscious patients, the drugs were dissolved in water delivered through a nasogastric tube and flushed afterwards. A rifampicin formulation of the same manufacturer has shown bioavailability in adults equal to the international reference.^17^ Patients were given adjunctive oral prednisone (2–4 mg/kg) for the first 4–8 weeks, tapered according to the national guidelines.^16^

### PK assessments

PK sampling was performed on days 2±1 and 10±1 of treatment. Serial venous blood samples were collected at 0, 1, 2, 4 and 8 hours postdose; one CSF sample was also collected at 0–2, 3–5 or 6–8 hours postdose. Patients had an overnight fast from 23:00 hours on the day preceding PK assessments until 2 hours after drug administration. Bioanalysis was performed using a validated ultra-performance liquid chromatography method.^18^ PK parameters were assessed non-compartmentally using the PKNCA package V.0.9.4 in R for Windows. Main PK parameters were area under the plasma concentration–time curve during the daily dosing interval (AUC_0–24_), peak plasma concentration (*C*_max_) and CSF concentration (*C*_CSF0–8_). Further details are given in online supplemental appendix 1.

10.1136/archdischild-2020-321426.supp1 Supplementary data

### Follow-up and clinical responses

Inpatient assessments were performed on days 3, 7, 10 and 14 of treatment, including physical examinations, Glasgow Coma Scale, anthropometry, vital signs and complications such as hyponatraemia, decreased consciousness, new focal neurological signs and suspicion of DILI. Additional assessments were performed, if necessary. Liver function tests (LFTs) were measured on days 7 and 14 of treatment and were subsequently measured if symptomatic DILI was suspected. DILI was defined as an elevation of ALT/AST>3× the ULN with symptoms of hepatotoxicity (eg, jaundice, vomiting, nausea and abdominal pain) or >5× the ULN without the presence of symptoms.^19^ The severity of DILI was classified based on the common terminology criteria for adverse events (CTCAE V.5.0; https://evs.nci.nih.gov/ftp1/CTCAE). Outcome of hospitalisation included good recovery, moderate and severe disabilities, persistent vegetative state and death. Six-month mortality was monitored by phone calls.

### Statistical analysis

On the basis of the results from a previous study in adult patients with TBM,^20^ a minimum of 20 patients were judged to be sufficient to describe PK of anti-TB drugs. Actual target values for rifampicin AUC_0–24_ in TBM (171 or 229 hour∙mg/L) were based on a PK/PD analysis in Indonesian adults with TBM,^21^ and the proportion of patients achieving these target values was assessed. PK parameters on both sampling days were compared using a paired-sample t-test or Wilcoxon signed-rank test. Pearson correlation coefficients were used to assess the relationship between AUC_0–24_, *C*_max_ and *C*_CSF0–8_. Predictors of drug exposures were evaluated using univariate and multivariate linear regression analyses; more details are given in online supplemental appendix 2. AUC_0–24_ and *C*_max_ values between DILI and non-DILI patients and between those who survived and died during the 6-month follow-up were compared using the Mann-Whitney U test. Data were analysed using SPSS Statistics (V.25.0; IBM).

## Results

Between March 2018 and July 2019, 81 suspected cases of paediatric TBM (39 (48%) aged <5 years) were screened, of whom 61 were excluded due to various reasons (online supplemental appendix 3). Among 20 eligible HIV-negative patients with probable/possible TBM, 11 (55%) were female, 5 (25%) aged <5 years and 12 (60%) had grade 2 TBM. Baseline characteristics of the study population are presented in table 1.

**Table 1:** Baseline characteristics and drug doses of Indonesian children with TBM

Characteristics	Value
Total cases, n	20
Female sex (n (%))	11 (55.0)
Age, years (median (IQR))	11.4 (4.4–14.7)
Age (n (%))	
<5 years	5 (25.0)
5–9 years	4 (20.0)
10–14 years	6 (30.0)
15–18 years	5 (25.0)
BCG-vaccinated (n (%))	11 (55.0)
Nutritional status*	
Weight for age Z-score (median (IQR))†	−2.08 (−3.06 to −1.32)
Height for age Z-score (median (IQR))	−2.10 (−2.44 to −1.21)
BMI for age Z-score (median (IQR))	−2.22 (−2.98 to −1.02)
Head circumference, cm (median (IQR))	50.0 (45.6–52.2)
Upper arm circumference, cm (median (IQR))	16.2 (12.6–20.6)
Abdominal circumference, cm (median (IQR))	52.5 (46.7–58.2)
Malnourished, n (%)	14 (70.0)
Temperature, °C (median (IQR))	37.1 (37.0–37.8)
Chief complaint (n (%))	
Severe headache	3 (15.0)
Seizures	4 (20.0)
Decreased consciousness	9 (45.0)
Others	4 (20.0)
Diagnostic score (median (IQR))‡	10.5 (10.0–12.0)
GCS (median (IQR))	13.0 (11.0–15.0)
Chest radiography, suggestive TB (n (%))	8 (40.0)
TBM category	
Possible TBM	2 (10.0)
Probable TBM	18 (90.0)
TBM grade (n (%))§	
Grade 1	4 (20.0)
Grade 2	12 (60.0)
Grade 3	4 (20.0)
CSF baselines (median (IQR))	
Leucocytes, cells/µL	88.0 (41.0–134.2)
PMN, cells/µL	20.5 (5.0–43.7)
MN, cells/µL	79.5 (56.2–95.0)
Protein, mg/dL	176.9 (80.7–287.5)
CSF/blood glucose ratio (median (IQR))	0.17 (0.10–0.44)
CSF smear microscopy (n (%))	
Negative	15 (75.0)
Not tested	5 (25.0)
Cerebral imaging, done (n (%))¶	12 (60.0)
Abnormal	11 (55.0)
Hydrocephalus	7 (35.0)
Basal meningeal enhancement	4 (20.0)
Brain oedema	4 (20.0)
Midline shift	2 (10.0)
Tuberculoma	1 (5.0)
Infarct	1 (5.0)
Intracerebral haemorrhage	1 (5.0)
Normal	1 (5.0)
GeneXpert MTB/RIF testing (extraneural), done (n (%))**	4 (20.0)
M.*tb* detected, susceptible to rifampicin	3 (15.0)
M.*tb* not detected	1 (5.0)
Blood test values (median (IQR))	
Creatinine, mg/dL	0.5 (0.3–0.6)
Albumin, g/dL	3.2 (2.4–3.5)
Protein, g/dL	7.6 (6.9–8.4)
Random blood glucose, mg/dL	107.0 (102.0–119.0)
AST, IU/L	22.0 (16.0–33.0)
ALT, IU/L	16.0 (13.0–30.0)
Drug administration through NGT on PK1 (n (%))	14 (70.0)
Drug administration through NGT on PK2 (n (%))	4/12 (20.0)
Daily drug doses on PK1 (median (IQR))	
Isoniazid (mg/kg)	8.9 (7.7–11.0)
Rifampicin (mg/kg)	13.4 (11.6–16.4)
Pyrazinamide (mg/kg)	26.7 (23.1–32.9)
Ethambutol (mg/kg)	20.5 (19.1–21.6)

*Anthropometric data were transformed into weight-for-age, height-for-age and BMI-for-age Z-scores based on the WHO standard reference populations using the R package ‘zscorer’ V.0.3.1. Malnutrition was defined as children aged <5 years with weight-for-age or height-for-age Z-scores <−2 SD and children aged ≥5 years with height-for-age or BMI-for-age Z-scores <−2 SD.

†Weigh for age Z-score can only be calculated for nine children.

‡Diagnostic score was assessed using a uniform case definition criteria for TBM by Marais *et al*.^15^

§Severity of TBM was classified according to the modified British Medical Research Council grading system as 1 (GCS of 15 with no focal neurological signs), 2 (GCS of 11–14 or 15 with focal neurological signs) or 3 (GCS<10).^44^

¶During hospitalisation, head computed tomographic scan was performed in 11 (55%) of 20 patients and head magnetic resonance imaging was performed in 1 (5%) of 20 patients.

**Three patients were susceptible to rifampicin using GeneXpert testing from gastric lavage sample, and one patient had no *M. tb* detected in sputum sample.

ALT, alanine aminotransferase; AST, aspartate aminotransferase; BMI, body mass index; CSF, cerebrospinal fluid; GCS, Glasgow Coma Scale; MN, mononuclear cells; NGT, nasogastric tube; PK1 and PK2, first and second pharmacokinetic sampling assessments; PMN, polymorphonuclear cells; TBM, tuberculous meningitis.

**Figure 1:**
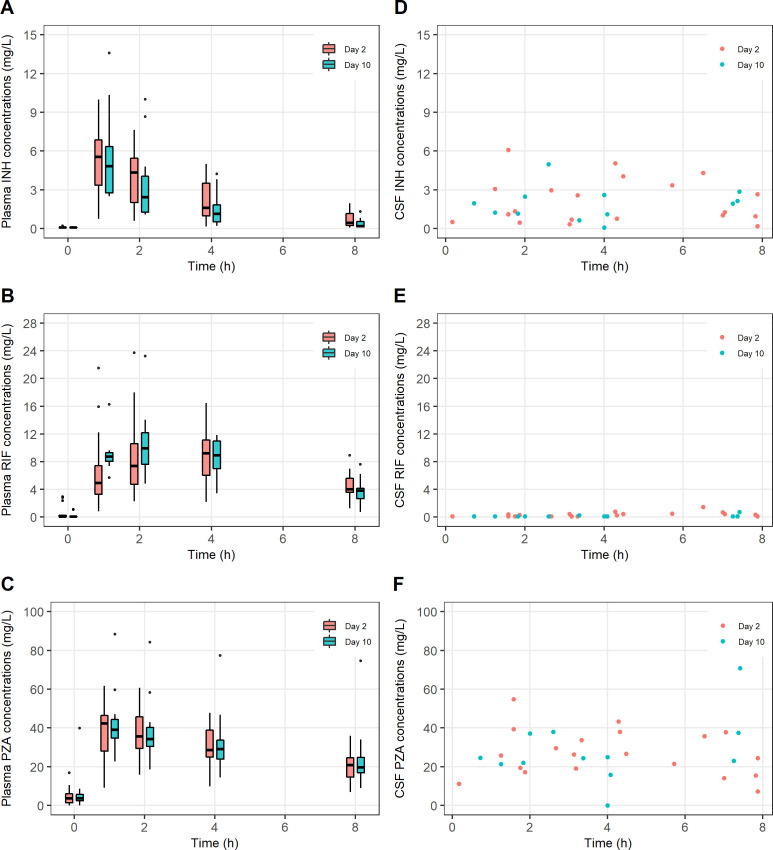
Pharmacokinetic profiles (drug concentration vs time curves) of isoniazid (INH), rifampicin (RIF) and pyrazinamide (PZA) in children and adolescents treated for tuberculous meningitis on days 2 and 10 of treatment. (A) INH in plasma; (B) RIF in plasma; (C) PZA in plasma; (D) INH in cerebrospinal fluid (CSF); (E) RIF in CSF; (F) PZA in CSF.

Plasma concentration–time profiles of isoniazid, rifampicin and pyrazinamide are presented in figure 1. Geometric mean AUC_0–24_ of isoniazid, rifampicin and pyrazinamide on day 2 was 18.5, 66.9 and 315.5 hour∙mg/L, respectively. Large interindividual variabilities were observed in AUC_0–24_ and *C*_max_ of all drugs. None of the patients had achieved the target values of 229 or 171 hour∙mg/L for rifampicin AUC_0–24_. All patients had disproportionately lower rifampicin concentrations in CSF than in plasma (geometric mean *C*_CSF0–8_: 0.3 and 0.1 mg/L on days 2 and 10, respectively). Isoniazid and pyrazinamide concentrations in CSF were relatively comparable to those in plasma. AUC_0–24_ and *C*_max_ between both sampling days were not statistically different (table 2). Additional PK parameters are presented in online supplemental appendix 4.

**Table 2:** Summary of pharmacokinetic (PK) parameters of isoniazid, rifampicin and pyrazinamide among Indonesian children treated for TBM

PK parameters	First PK assessment (n=20)	Second PK assessment (n=12)	P value*
Isoniazid			
AUC_0–24_ (h∙mg/L)	18.5 (5.1–47.4)	14.5 (5.9–44.2)	0.888
*C*_max_ (mg/L)	4.6 (1.0–10.0)	4.7 (2.5–13.6)	0.366
*C*_CSF0–2_ (mg/L)†	1.4 (0.5–6.1)	1.6 (1.2–2.5)	n/a
*C*_CSF3–5_ (mg/L)†	1.6 (0.3–5.0)	1.7 (0.6–5.0)	n/a
*C*_CSF6–8_ (mg/L)†	1.3 (1.2–4.3)	2.3 (1.9–2.8)	n/a
Rifampicin			
AUC_0–24_ (h∙mg/L)	66.9 (21.7–118.6)	71.8 (36.1–116.5)	0.442
*C*_max_ (mg/L)	9.4 (2.9–23.7)	10.4 (5.7–23.3)	0.499
*C*_CSF0–2_ (mg/L)†	0.2 (0.1–0.4)	0.1 (0.1–0.1)	n/a
*C*_CSF3–5_ (mg/L)†	0.3 (0.1–0.8)	0.1 (0.1–0.3)	n/a
*C*_CSF6–8_ (mg/L)†	0.4 (0.1–1.4)	0.2 (0.1–0.7)	n/a
Pyrazinamide			
AUC_0–24_ (h∙mg/L)	315.5 (100.6–599.0)	328.4 (143.3–1477.7)	0.482
*C*_max_ (mg/L)	37.7 (15.9–61.7)	40.5 (22.7–88.4)	0.350
*C*_CSF0–2_ (mg/L)†	24.4 (11.1–54.9)	25.6 (21.3–37.1)	n/a
*C*_CSF3–5_ (mg/L)†	30.0 (19.2–43.3)	24.7 (15.9–38.1)	n/a
*C*_CSF6–8_ (mg/L)†	19.6 (7.2–37.7)	39.4 (23.1–70.8)	n/a

Data are presented as geometric mean (range). The first PK assessment was performed on day 2 of treatment and the second PK assessment was performed on day 10 of treatment.

*Paired-sample t-test on log-transformed data of 12 patients for whom PK data were available both at the first and second PK assessments.

†At the first PK assessment, 6, 7 and 7 CSF samples for each drug were available at 0–2 hours, 3–5 hours and 6–8 hours, respectively; and at the second PK assessment, 4, 4 and 3 CSF samples for each drug were available at 0–2 hours, 3–5 hours and 6–8 hours, respectively.

AUC_0–24_, area under the plasma concentration–time curve from 0 to 24 hours postdose; *C*_CSF0–8_, drug concentration in cerebrospinal fluid during 0–8 hours postdose; *C*_max_, peak plasma concentration; n/a, non-applicable; TBM, tuberculous meningitis.

For each drug, AUC_0–24_ was highly correlated with *C*_max_ (*r_s_* ≥0.7; p<0.001). AUC_0–24_ and *C*_max_ were also correlated with *C*_CSF0–8_ (*r_s_* ≥0.5; p<0.05) (online supplemental appendix 5. Results of the univariate analyses for predictors of AUC_0–24_, *C*_max_ and *C*_CSF0–8_ are presented in online supplemental appendix 6. In multivariate analyses, higher drug doses in mg/kg were associated with a larger increase in pyrazinamide *C*_max_ (p<0.05); drug administration through a nasogastric tube was associated with a higher isoniazid AUC_0–24_ (p<0.01); and higher random blood glucose levels were associated with reduced pyrazinamide AUC_0–24_, *C*_max_ and *C*_CSF0–8_ (p<0.01) (table 3).

**Table 3:** Multivariate linear regression analysis of factors associated with AUC_0–24_, *C*_max_ and CSF concentrations of isoniazid, rifampicin and pyrazinamide in Indonesian children treated for TBM

	AUC_0–24,_ hour∙mg/L (B (95% CI))	*C*_max_, mg/L (B (95% CI))	*C*_CSF_0–8, mg/L (B (95% CI))
Isoniazid			
Age, years	n/a	−0.020 (−0.043 to 0.003)^#^	n/a
Random blood glucose, mg/dL	−0.002 (−0.006 to 0.003)	−0.004 (−0.009 to 0.001)	−0.007 (−0.015 to 0.001)^#^
Drug dose, mg/kg	0.016 (−0.048 to 0.080)	n/a	0.046 (−0.058 to 0.151)
Drug administration via NGT, no/yes	0.439 (0.143 to 0.735)**	0.130 (−0.160 to 0.420)	0.289 (−0.197 to 0.775)
Rifampicin			
Age, years	−0.009 (−0.028 to 0.010)	−0.008 (−0.029 to 0.012)	−0.021 (−0.052 to 0.009)
Random blood glucose, mg/dL	−0.003 (−0.007 to 0.001)	−0.005 (−0.009 to −0.0003)*	n/a
Drug dose, mg/kg	0.014 (−0.021 to 0.048)	n/a	0.030 (−0.030 to 0.091)
Drug administration via NGT, no/yes	n/a	0.067 (−0.194 to 0.328)	0.019 (−0.365 to 0.403)
Pyrazinamide			
Random blood glucose, mg/dL	−0.006 (−0.010 to −0.003)**	−0.003 (−0.005 to −0.001)**	−0.006 (−0.010 to −0.003)**
Drug dose, mg/kg	0.010 (−0.006 to 0.027)	0.010 (0.001 to 0.020)*	0.010 (−0.006 to 0.027)
Drug administration via NGT, no/yes	−0.068 (−0.293 to 0.156)	0.036 (−0.095 to 0.167)	−0.068 (−0.293 to 0.156)

Data are presented as regression coefficients (B) and 95% CIs. ^#^p<0.1, *p<0.05, **p<0.01.

The total explained variance (R^2^) for isoniazid AUC_0–24_: 0.57, isoniazid *C*_max_: 0.46, isoniazid *C*_CSF0–8_: 0.45; rifampicin AUC_0–24_: 0.31, rifampicin *C*_max_: 0.38, rifampicin *C*_CSF0–8_: 0.33, pyrazinamide AUC_0–24_: 0.53, pyrazinamide *C*_max_: 0.63 and pyrazinamide *C*_CSF0–8_: 0.53.

AUC_0–24_, area under the plasma concentration–time curve from 0 to 24 hours postdose at the first PK assessment; *C*_CSF0–8_, CSF concentrations during 0–8 hours postdose at the first PK assessment; CI, confidence interval; *C*_max_, peak plasma concentration at the first PK assessment; n/a, non-applicable; NGT, nasogastric tube; TBM, tuberculous meningitis.

**Figure 2:**
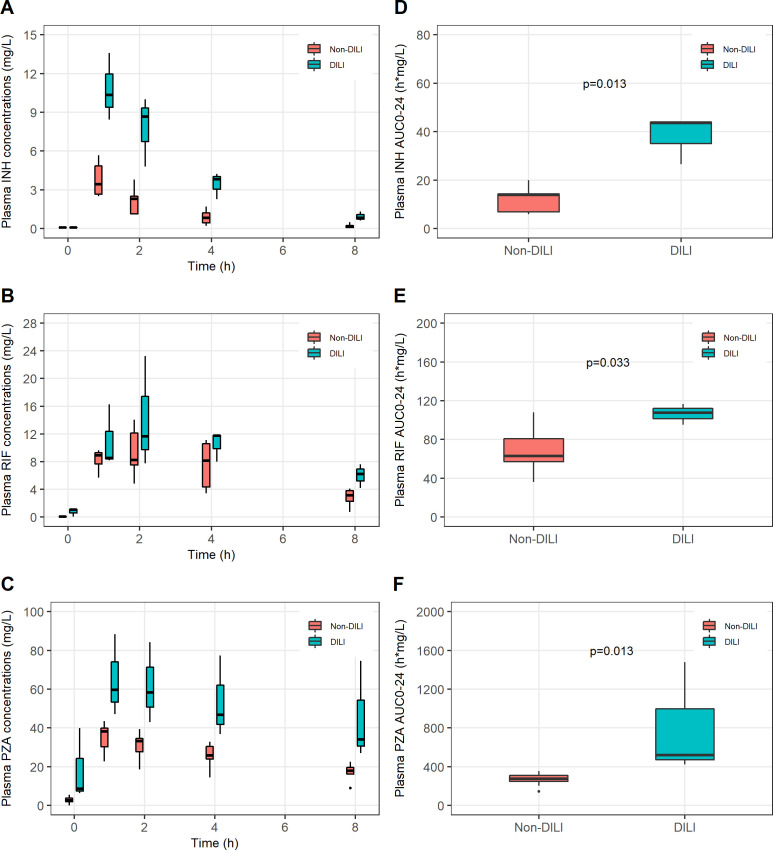
Pharmacokinetic profiles of isoniazid (INH), rifampicin (RIF) and pyrazinamide (PZA) on day 10 of tuberculous meningitis treatment in children and adolescents who developed antituberculosis drug-induced liver-injury (DILI, n=3*) and those without DILI (n=9). (A) INH plasma concentration vs time curve; (B) RIF plasma concentration vs time curve; (C) PZA plasma concentration vs time curve; (D) INH area under the concentration–time curve during the dosing interval (AUC_0–24_); (E) RIF AUC_0–24_; (F) PZA AUC_0–24_. Box plots represent medians with IQRs; lower and upper whiskers represent first and fourth quartiles, respectively. *Of four patients with DILI, one who developed DILI on day 7 of treatment did not have INH, RIF and PZA concentrations measured on day 10 because the drugs had been temporarily stopped due to DILI.

During hospitalisation, two patients had grade 2 and two patients had grade 3 DILI. Of these, one developed DILI after 1 week of treatment, two after 2 weeks and one after 4 weeks. Jaundice was observed in two patients: one with grade 2 and one with grade 3 DILI. Isoniazid, rifampicin and pyrazinamide were immediately stopped in these four patients. As per local guidelines, ethambutol was continued, with addition of streptomycin (15–40 mg/kg) for a maximum of 2 weeks. After the symptoms of DILI and liver enzymes had normalised, rifampicin and isoniazid were reintroduced gradually without any DILI recurrence. Pyrazinamide was completely stopped until the end of treatment. Isoniazid, rifampicin and pyrazinamide doses were slightly higher in patients with DILI but not statistically different from those without DILI (p>0.05; online supplemental appendix 7). AUC_0–24_ of isoniazid, rifampicin and pyrazinamide, along with *C*_max_ of isoniazid and pyrazinamide on day 10 were significantly higher in patients with DILI than those without DILI (p<0.05) (figure 2; online supplemental appendix 7).

At hospital discharge, 10 patients had good recovery, 2 were moderately disabled, 2 were severely disabled and 6 died due to increased intracranial pressure (n=2), intracerebral haemorrhage (n=1), septic shock (n=1), respiratory failure (n=1) and hospital-acquired pneumonia (n=1). Within the 6-month follow-up, another patient died of unknown cause and the remaining 13 patients survived including those who previously developed DILI. Post-mortem autopsy was unavailable to provide accurate causes of death. AUC_0–24_, *C*_max_ and *C*_CSF0–8_ of isoniazid, rifampicin and pyrazinamide were not statistically different between patients who survived and died within the 6-month follow-up.

## Discussion

This study presents important information on plasma and CSF concentrations of first-line anti-TB drugs in children and adolescents with TBM from Indonesia. Our average AUC_0–24_ values on day 2 of treatment compared with those reported in Indonesian adults with TBM were relatively similar for isoniazid (18.5 vs 16.4 hour∙mg/L),^22^ were higher for rifampicin (66.9 vs 53.5 hour∙mg/L)^20^ and were lower for pyrazinamide (315.5 vs 709 hour∙mg/L).^23^ Our results showed large interindividual variabilities in drug exposures, which are in agreement with the literature and might be enhanced by PK changes in critically ill patients.^24 25^ Furthermore, the wide age range included in this study from infants to adolescents, and the small sample size, might contribute to these large variabilities. Although none of our patients had diabetes mellitus, higher blood glucose levels were found to be associated with decreased pyrazinamide exposures. A hyperglycaemic condition may have reduced gastric mucosal blood flow and gastric acid secretion,^26^ which resulted in decreased absorption of anti-TB drugs.

The low rifampicin CSF concentration has been reported in Vietnamese children^11^ and in Indonesian adults with TBM.^18 20 27^ Likely the high plasma protein binding and blood-CSF/brain barrier efflux pumps can explain this low CSF rifampicin concentration.^28^ The bactericidal effect of such a low concentration, when compared with the minimum inhibitory concentration (MIC) of this drug against *M. tuberculosis*, is likely to be limited if we use plasma-derived *f*AUC/MIC or *fC*_max_/MIC targets. In adults, a 33% higher dose of intravenous rifampicin resulted in a threefold increase in plasma and CSF exposures compared with the standard dose of oral rifampicin.^27^ Threefold and fivefold increases in plasma exposure with proportional increases in CSF concentrations were also observed in adults given double and triple doses of oral rifampicin.^20^ It seems that efflux pumps may be saturable and CSF rifampicin concentrations can be enhanced by increasing the dose. In South African children, short-course intensified TBM treatment with isoniazid (20 mg/kg), rifampicin (20 mg/kg), pyrazinamide (40 mg/kg) and ethionamide (20 mg/kg) was found to be safe and effective.^12^ Intensified regimens containing high-dose rifampicin and other anti-TB drugs with better CSF penetration (eg, fluoroquinolones and ethionamide), along with ancillary treatment beyond corticosteroids such as targeted anti-inflammatory drugs (eg, aspirin, thalidomide and tumour necrosis factor-alpha antibodies), need further evaluation.^12 29^

Serious adverse events in children during TB treatment are rare although severe hepatotoxic events were occasionally reported.^14 30^ In a review by Donald,^14^ abnormal LFTs and jaundice were recorded, respectively, in 53% and in 10% of children during TBM therapy. In Indonesian settings, DILI frequently occurred in children during the first 2 months of TB therapy,^31 32^ with most of them being treated for TBM.^31^ The reason why patients with TBM are more likely to develop DILI is unclear but could be related to the severity of the underlying disease.^33^ Of note, this hepatotoxic event could have been the result of hepatic adaptation.^19^ The temporary use of streptomycin in this study could not be regarded as an effective treatment.^10 34^ Better management of DILI in children (including criteria to continue treatment in severe conditions and drug reintroduction regimens) is needed.

Data on the relationship between DILI and anti-TB drug exposures in children are lacking.^14^ In Chinese and Indian adults with PTB/extrapulmonary TB, higher isoniazid and rifampicin exposures were associated with an increased risk of DILI.^35 36^ High-dose rifampicin was not associated with an increase in DILI when administered to Tanzanian and South-African adults with PTB.^37^ In Indonesian adult patients with TBM, hepatotoxicity was not related to rifampicin exposure^20 27^ and was equally distributed between rifampicin standard-dose and high-dose groups.^18 20 27^ It should be acknowledged that neither the current study nor the previous studies in Indonesian adults^18 20 27^ were powered to test for an association between drug levels and DILI. Our findings on DILI in children with TBM warrant further investigation as DILI has clinical implications in increasing patient morbidity/mortality.^38^ The risk of DILI with increased drug dosages requires consideration, but it should be balanced against the need to ensure optimal treatment of a life-threatening illness like TBM.^29 39^ Combining therapeutic drug monitoring as a decisive tool for TB treatment,^40^ and regular monitoring of LFTs,^19^ might benefit to ensure drug efficacy without causing toxicity.

Our study has some limitations. Conducting intensive PK studies in children is challenging. Ideally, multiple CSF samples are required to assess CSF-to-plasma ratio for total drug exposure.^41^ Although we were able to collect two PK curves with a modest number of plasma samples, only one CSF sample per patient could be collected. As a result, AUC_0–24_ and *C*_max_ in CSF could not be determined. None of our patients had definite TBM because mycobacterial confirmation is known to be a significant challenge in children, due to the paucibacillary nature of the disease and low CSF volumes available for diagnostic analysis.^42^ Relatively few young children who may be considered most at risk for being underdosed were included in this study. Our results pointed out, however, no difference in drug exposure between younger and older children. Of note, our population was not representative of the total paediatric TBM patients diagnosed over the study period. Due to the small sample size, our findings on predictors of exposure to anti-TB drugs and the relationship of drug exposures with DILI and 6-month mortality should be interpreted with caution. It could be of value to collect data on drug exposure and pathogen susceptibility in a large cohort to overcome the limitations of small-scale PK studies.^43^

In conclusion, suboptimal plasma and CSF rifampicin concentrations were observed in all patients, and there is an urgent need to increase the rifampicin dose in children and adolescents with TBM. Intensified regimens containing anti-TB drugs with better CSF penetration, along with other ancillary treatment for paediatric TBM, warrant further evaluation. The association between higher isoniazid, rifampicin and pyrazinamide concentrations and the development of DILI needs confirmatory studies.

## Data Availability

The datasets used and/or analysed during the current study are available from the corresponding author on reasonable request.
