# Supplementary material for: Pharmacokinetics and safety/tolerability of isoniazid, rifampicin and pyrazinamide in children and adolescents treated for tuberculous meningitis
Source: Arch Dis Child. 2021 Jun 28;107(1):70–7. doi: 10.1136/archdischild-2020-321426 (PMC8685623; doi:10.1136/archdischild-2020-321426)
Supplement: Supplementary data [file archdischild-2020-321426supp001.pdf]

Supplementary material:

Pharmacokinetics and safety/tolerability of isoniazid, rifampicin and pyrazinamide in children and adolescents treated for tuberculous meningitis

Rovina Ruslami,[1,¶] Fajri Gafar,[2,¶,*] Vycke Yunivita,[1] Ida Parwati,[3] Ahmad R. Ganiem,[4] Rob E. Aarnoutse,[5] Bob Wilffert,[2,6] Jan-Willem C. Alffenaar,[7,8] Heda M. Nataprawira[9]

[1] Universitas Padjadjaran, Faculty of Medicine, Department of Biomedical Sciences, Division of Pharmacology and Therapy, Bandung, Indonesia; [2] University of Groningen, Groningen Research Institute of Pharmacy, Unit of PharmacoTherapy, -Epidemiology, and -Economics, Groningen, the Netherlands; [3] Universitas Padjadjaran, Hasan Sadikin Hospital, Faculty of Medicine, Department of Clinical Pathology, Bandung, Indonesia; [4] Universitas Padjadjaran, Hasan Sadikin Hospital, Faculty of Medicine, Department of Neurology, Bandung, Indonesia; [5] Radboud University Medical Center, Radboud Institute for Health Sciences, Department of Pharmacy, Nijmegen, The Netherlands; [6] University of Groningen, University Medical Center Groningen, Department of Clinical Pharmacy and Pharmacology, Groningen, the Netherlands; [7] University of Sydney, Faculty of Medicine and Health, School of Pharmacy, Sydney, Australia; [8] Westmead Hospital, Sydney, Australia; and [9] Universitas Padjadjaran, Hasan Sadikin Hospital, Faculty of Medicine, Department of Child Health, Division of Pediatric Respirology, Bandung, Indonesia.

[¶]These first authors contributed equally to this manuscript

***Corresponding author:**

Fajri Gafar; University of Groningen, Groningen Research Institute of Pharmacy, Unit of PharmacoTherapy, -Epidemiology and -Economics, Antonius Deusinglaan 1 (room: 3214.0450), 9713 AV Groningen, The Netherlands, E-mail: f.gafar@rug.nl

Ruslami R, *et al. Arch Dis Child* 2021; 107:70–77. doi: 10.1136/archdischild-2020-321426

Appendix-1. PK assessments

On both sampling days, blood and CSF samples were collected for each patient in EDTA-coated tubes, placed immediately on ice, centrifuged at 3000 rpm for 15 min, and stored at -80 °C within 30 min after sample collection. Bioanalysis was performed at the Pharmacokinetic Laboratory of the Faculty of Medicine of Universitas Padjadjaran, using an ultra-performance liquid chromatography method [1]. The accuracy for plasma and CSF assays ranged, respectively, from 101.7-109.0% and 97.1-103.0% for isoniazid, 95.1-102.4% and 94.5-100.7% for rifampicin, and 99.1-102.1% and 85.8-95.5% for pyrazinamide, depending on the concentration level. Intraday and interday coefficients of variation were <7.9% and <8.1% over the 0.15-15 mg/L concentration range for isoniazid in plasma and CSF, <4.2% and <3.4% over the concentration ranges of 0.125-30 mg/L and 0.25-30 mg/L for rifampicin in plasma and CSF, and <3.9% and <6.6% over the 0.20-60.06 mg/L concentration range for pyrazinamide in plasma and CSF, respectively.

PK parameters were assessed noncompartmentally using the R package "PKNCA" ver.0.9.4 in R for Windows. Drug concentrations below the lower limit of quantification (LLOQ) were set to half of the LLOQ. Main PK measures were area under the plasma concentration-time curve during the dosing interval ($AUC_{0\text{-}24}$), peak plasma concentration (C_{max}) and CSF concentration ($C_{CSF0\text{-}8}$). C_{max} and the corresponding time to C_{max} (T_{max}), were derived directly from the concentration-time curves. Plasma concentration at 24 h post-dose was calculated with the formula: $C_{24}=C_{last}\times e^{-\beta\times(24-T_{last})}$, in which C_{last} is the last measurable concentration at T_{last}, and β is the first order elimination rate constant. β was obtained by least-squares linear regression analysis on log concentration versus time, with the absolute slope of the regression line being $\beta/2.303$. The terminal log-linear phase was determined by the last three data points; if the last three points were not available, only the last two points were used. The elimination half-life ($t_{1/2}$) was obtained by the equation: $0.693/\beta$. $AUC_{0\text{-}24}$ was calculated based on the linear-up/log-down trapezoidal rule. Apparent clearance (CL/F) was obtained by dividing the dose by $AUC_{0\text{-}24}$, and apparent volume distribution (V_d/F) was obtained by dividing CL/F by β.

Ruslami R, *et al. Arch Dis Child* 2021; 107:70–77. doi: 10.1136/archdischild-2020-321426

Appendix-2. Statistical analysis

Predictors of drug exposures were evaluated using univariate and multivariate linear regression analyses. Variables in the univariate analysis showing a trend towards association ($p<0.25$) with each of the above dependent PK variables were eligible for inclusion in a multivariate analysis. Due to the small sample size of patients, we only allowed a maximum of three variables to be included in the multivariate models. The models having the highest total explained variance (R^2) and preferably including the highest number of patients were selected as the final models.

Ruslami R, *et al. Arch Dis Child* 2021; 107:70–77. doi: 10.1136/archdischild-2020-321426

Appendix-3. Selection of study participants.

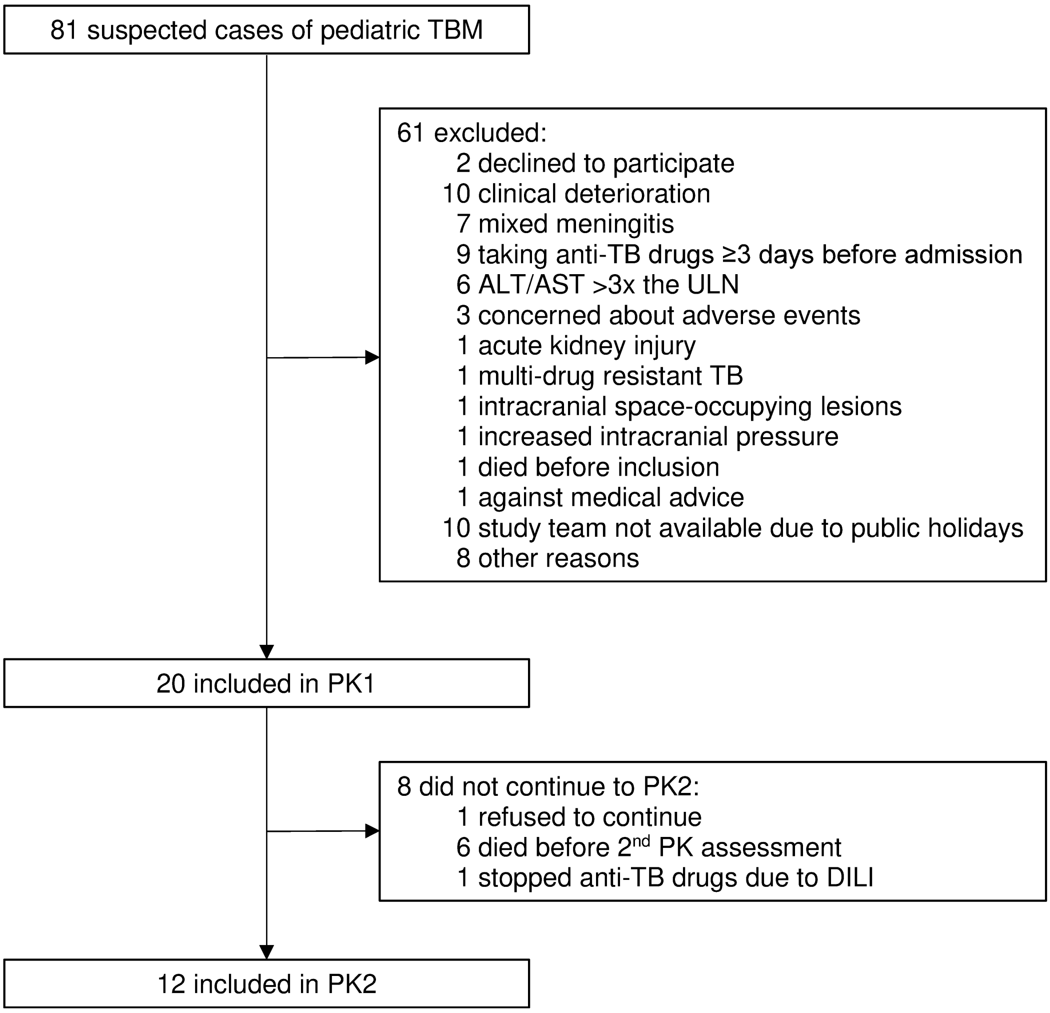

Note: TB: tuberculosis; TBM: tuberculous meningitis; PK1: first pharmacokinetic assessment on day 2 of treatment; PK2: second pharmacokinetic assessment on day 10 of treatment; ALT: alanine aminotransferase; AST: aspartate aminotransferase; DILI: drug-induced liver injury due to anti-TB drugs

Appendix-4. Additional pharmacokinetic (PK) parameters of isoniazid, rifampicin and pyrazinamide among Indonesian children treated for TBM.

PK parameters	1st PK assessment (n=20)	2nd PK assessment (n=12)	p-value
Isoniazid			
T_{max} (h)[a]	1.0 (1.0-1.9)	1.0 (1.0-1.0)	0.107[c]
CL/*F* (L/h)	9.8 (1.1-59.0)	14.0 (4.6-42.2)	0.888[b]
V_d/F (L)	11.9 (2.1-63.7)	13.8 (4.8-35.8)	0.815[b]
$t_{1/2}$ (h)	0.8 (0.3-2.0)	0.7 (0.3-1.0)	0.945[b]
Rifampicin			
T_{max} (h)[a]	4.0 (2.0-4.0)	2.0 (1.0-3.5)	0.015[c]
CL/*F* (L/h)	4.1 (0.9-20.7)	4.2 (2.4-11.2)	0.442[b]
V_d/F (L)	11.2 (1.7-52.9)	12.2 (2.6-56.0)	0.973[b]
$t_{1/2}$ (h)	1.9 (1.0-10.0)	2.0 (0.7-6.2)	0.656[b]
Pyrazinamide			
T_{max} (h)[a]	1.0 (1.0-2.0)	1.0 (1.0-1.0)	0.196[c]
CL/*F* (L/h)	1.7 (0.2-8.9)	1.8 (0.4-5.2)	0.482[b]
V_d/F (L)	8.6 (2.5-29.2)	9.2 (3.6-18.8)	0.614[b]
$t_{1/2}$ (h)	3.5 (1.8-7.6)	3.4 (1.5-16.2)	0.592[b]

Data are presented as geometric mean (range), unless otherwise stated [a]:median (interquartile range). The first PK assessment was performed on day 2 of treatment, and the second PK assessment was performed on day 10 of treatment. T_{max}: time to peak plasma concentration; CL/*F*: apparent total clearance; V_d/F: apparent volume distribution; $t_{1/2}$: elimination half-life; TBM: tuberculous meningitis. [b]:Paired-sample t-test on log-transformed data of 12 patients for whom PK data were available both at the first and second PK assessments [c]:Wilcoxon signed-rank test between the first and second PK assessments.

Ruslami R, *et al. Arch Dis Child* 2021; 107:70–77. doi: 10.1136/archdischild-2020-321426

Appendix-5. Correlations between $AUC_{0\text{-}24}$, C_{max} and $C_{CSF0\text{-}8}$ at the first and second PK assessments of Indonesian children treated for TBM.

	Isoniazid		Rifampicin		Pyrazinamide	
	r	p-value	*r*	p-value	*r*	p-value
1st PK assessment (day 2)						
$AUC_{0\text{-}24}$ vs. C_{max}	0.83	<0.001	0.80	<0.001	0.89	<0.001
$AUC_{0\text{-}24}$ vs. $C_{CSF0\text{-}8}$	0.69	0.001	0.49	0.028	0.77	<0.001
C_{max} vs. $C_{CSF0\text{-}8}$	0.59	0.007	0.60	0.005	0.72	<0.001
2nd PK assessment (day 10)						
$AUC_{0\text{-}24}$ vs. C_{max}	0.96	<0.001	0.69	0.014	0.91	<0.001
$AUC_{0\text{-}24}$ vs. $C_{CSF0\text{-}8}$	0.88	<0.001	0.50	0.121	0.92	<0.001
C_{max} vs. $C_{CSF0\text{-}8}$	0.80	0.003	0.17	0.611	0.91	<0.001

Data are presented as Pearson correlation coefficient (*r*). $AUC_{0\text{-}24}$: area under the plasma concentration-time curve from 0-24 h post-dose; C_{max}: peak plasma concentration; $C_{CSF0\text{-}8}$: cerebrospinal fluid concentration during 0-8 h post dose; TBM: tuberculous meningitis.

Ruslami R, *et al. Arch Dis Child* 2021; 107:70–77. doi: 10.1136/archdischild-2020-321426

Appendix-6. Univariate linear regression analysis of factors associated with AUC_{0-24}, C_{max} and C_{CSF0-8} of isoniazid, rifampicin and pyrazinamide in Indonesian children treated for TBM.

	AUC_{0-24}, h·mg/L (B [95% CI])	C_{max}, mg/L (B [95% CI])	C_{CSF0-8}, mg/L (B [95% CI])
Isoniazid			
Age, years	-0.02 (-0.05; -0.002)*	-0.02 (-0.05; -0.003)*	-0.02 (-0.06; 0.01)
Sex, male/female	0.05 (-0.24; 0.35)	0.01 (-0.27; 0.28)	-0.04 (-0.45; 0.38)
[a]Malnourished, no/yes	0.29 (0.001; 0.57)*	0.10 (-0.20; 0.39)	0.41 (0.01; 0.82)*
Albumin, g/dL	-0.13 (-0.35; 0.10)	-0.16 (-0.38; 0.06)	-0.003 (-0.32; 0.32)
Random blood glucose, mg/dL	-0.004 (-0.01; 0.002)	-0.006 (-0.01; -0.0002)*	-0.01 (-0.02; -0.001)*
Creatinine clearance, mg/min	0.00 (-0.005l 0.006)	-0.001 (-0.01; 0.004)	0.00 (-0.01; 0.01)
TBM grade, 1/2/3	0.03 (-0.20; 0.26)	-0.003 (-0.22; 0.21)	-0.04 (-0.36; 0.29)
GCS score	-0.02 (-0.08; 0.05)	-0.001 (-0.06; 0.06)	-0.02 (-0.11; 0.07)
Drug dose, mg/kg	0.07 (0.001; 0.13)*	0.04 (-0.02; 0.11)	0.09 (-0.001; -0.19)#
Drug administration via NGT, no/yes	0.39 (0.14; 0.65)**	0.23 (-0.04; 0.51)#	0.44 (0.05; 0.84)*
Rifampicin			
Age, years	-0.01 (-0.03; 0.003)	-0.02 (-0.03; 0.003)	-0.03 (-0.05; -0.005)*
Sex, male/female	-0.06 (-0.25; 0.13)	-0.13 (-0.35; 0.10)	-0.06 (-0.39; 0.26)
[a]Malnourished, no/yes	0.11 (-0.09; 0.31)	0.08 (-0.17; 0.33)	-0.13 (-0.48; 0.21)
Albumin, g/dL	-0.06 (-0.19; 0.07)	-0.09 (-0.25; 0.07)	-0.09 (-0.35; 0.16)
Random blood glucose, mg/dL	-0.003 (-0.01; 0.0005)#	-0.006 (-0.01; -0.001)*	-0.003 (-0.01; 0.004)
Creatinine clearance, mg/min	-0.001 (-0.004; 0.003)	-0.003 (-0.01; 0.001)	-0.001 (-0.01; 0.005)
TBM grade, 1/2/3	-0.03 (-0.18; 0.12)	-0.02 (-0.20; 0.16)	0.05 (-0.20; 0.31)
GCS score	0.01 (-0.04; 0.05)	0.004 (-0.05; 0.06)	-0.04 (-0.11; 0.03)
Drug dose, mg/kg	0.02 (-0.01; 0.05)	0.02 (-0.02; 0.05)	0.05 (0.004; 0.10)*
Drug administration via NGT, no/yes	0.10 (-0.10; 0.30)	0.18 (-0.06; 0.41)	0.25 (-0.08; 0.58)
Pyrazinamide			
Age, years	-0.01 (-0.03; 0.001)#	-0.01 (-0.02; 0.00)#	-0.01 (-0.02; 0.01)
Sex, male/female	0.08 (-0.11; 0.28)	0.04 (-0.09; 0.18)	0.05 (-0.15; 0.26)
[a]Malnourished, no/yes	0.04 (-0.17; 0.25)	0.05 (-0.10; 0.19)	0.01 (-0.22; 0.24)
Albumin, g/dL	-0.12 (-0.24; 0.003)#	-0.07 (-0.16; 0.02)	-0.05 (-0.21; 0.09)
Random blood glucose, mg/dL	-0.005 (-0.01; -0.001)*	-0.004 (-0.01; -0.001)**	-0.006 (-0.01; -0.003)**
Creatinine clearance, mg/min	0.00 (-0.004; 0.003)	-0.001 (-0.003; 0.002)	0.00 (-0.004; 0.004)
TBM grade, 1/2/3	-0.03 (-0.19; 0.12)	-0.05 (-0.15; 0.06)	-0.06 (-0.23; 0.10)
GCS score	-0.01 (-0.05; 0.03)	0.01 (-0.02; 0.04)	-0.003 (-0.05; 0.04)
Drug dose, mg/kg	0.02 (0.002; 0.03)*	0.01 (0.005; 0.02)**	0.01 (-0.005; 0.03)
Drug administration via NGT, no/yes	0.20 (0.02; 0.39)*	0.15 (0.02; 0.28)*	0.10 (-0.12; 0.32)

Data are presented as regression coefficients (B) and 95% confidence intervals (CI); #p<0.1, *p<0.05, **p<0.01. AUC_{0-24}: area under the plasma concentration-time curve from 0-24 h post-dose at the first PK assessment (day 2 of treatment); C_{max}: peak plasma concentration at the first PK assessment; C_{CSF0-8}: CSF concentrations during 0-8 h post-dose at the first PK assessment; GCS: Glasgow comma scale; NGT: nasogastric tube; TBM: tuberculous meningitis. [a]: Malnutrition was defined as children aged <5 years with baseline weigh-for-age or height-for-age Z-scores <-2 standard deviations (SD), or children aged ≥5 years with baseline height-for-age or BMI-for-age Z-scores <-2 SD.

Appendix-7. Drug doses, AUC_{0-24} and C_{max} of isoniazid, rifampicin and pyrazinamide in patients who developed DILI and those without DILI during TBM treatment.

	DILI	Non-DILI	p-value*
1st PK assessment (day 2), n	4	16	
Isoniazid			
Dose (mg/kg)	10.6 (8.6-12.3)	8.8 (7.5-10.3)	0.185
AUC_{0-24} (h·mg/L)	27.1 (19.9-34.4)	16.8 (5.1-47.4)	0.299
C_{max} (mg/L)	6.4 (5.1-9.7)	4.3 (1.0-10.0)	0.450
Rifampicin			
Dose (mg/kg)	15.8 (12.9-18.5)	13.2 (11.2-15.4)	0.185
AUC_{0-24} (h·mg/L)	79.9 (55.9-114.8)	64.0 (21.7-118.6)	0.777
C_{max} (mg/L)	10.0 (7.2-16.5)	9.3 (2.9-23.7)	0.850
Pyrazinamide			
Dose	31.7 (25.8-36.9)	26.5 (22.5-30.9)	0.185
AUC_{0-24} (h·mg/L)	283.0 (198.7-575.4)	324.2 (100.6-599.0)	0.345
C_{max} (mg/L)	37.6 (26.3-54.2)	37.7 (15.9-61.7)	0.850
2nd PK assessment (day 10), n	3¶	9	
Isoniazid			
Dose (mg/kg)	11.8 (9.4-12.5)§	8.6 (7.1-9.7)	0.064
AUC_{0-24} (h·mg/L)	37.2 (26.6-44.2)	10.6 (5.9-19.9)	0.013
C_{max} (mg/L)	10.6 (8.4-13.6)	3.6 (2.5-5.7)	0.013
Rifampicin			
Dose (mg/kg)	17.6 (14.1-18.7)§	12.9 (10.7-14.6)	0.064
AUC_{0-24} (h·mg/L)	106.2 (95.4-116.5)	63.0 (36.1-108.0)	0.033
C_{max} (mg/L)	13.2 (8.5-23.3)	9.6 (5.7-14.1)	0.405
Pyrazinamide			
Dose (mg/kg)	35.3 (28.1-37.5)§	25.9 (21.4-29.2)	0.064
AUC_{0-24} (h·mg/L)	687.5 (423.6-1477.7)	256.7 (143.3-353.9)	0.013
C_{max} (mg/L)	62.8 (47.0-88.4)	34.9 (22.7-43.5)	0.013

Drug doses are presented as median (interquartile range), unless otherwise stated §:median (range). AUC_{0-24} and C_{max} values are presented as geometric mean (range). AUC_{0-24}: area under the plasma concentration-time curve from 0-24 h post-dose; C_{max}: peak plasma concentration; DILI: antituberculosis drug-induced liver injury; TBM: tuberculous meningitis. ¶: One patient who developed DILI on day 7 of treatment had no isoniazid, rifampicin and pyrazinamide concentrations measured at the second PK assessment as the drugs had been temporarily stopped due to DILI. *: Mann-Whitney U test between patients who developed DILI and those without DILI.

References:

[1] Yunivita V, Dian S, Ganiem AR, Hayati E, Hanggono Achmad T, Purnama Dewi A, et al. Pharmacokinetics and safety/tolerability of higher oral and intravenous doses of rifampicin in adult tuberculous meningitis patients. Int J Antimicrob Agents 2016;48:415–21.
